# The Actual Toxicity of Engine Exhaust Gases Emitted from Vehicles: The Development and Perspectives of Biological and Chemical Measurement Methods

**DOI:** 10.1021/acsomega.3c02171

**Published:** 2023-07-03

**Authors:** Aleksandra Kęska

**Affiliations:** Wroclaw University of Science and Technology, Department of Automotive Engineering, Wybrzeże Wyspiańskiego 27, 50-370 Wrocław, Poland

## Abstract

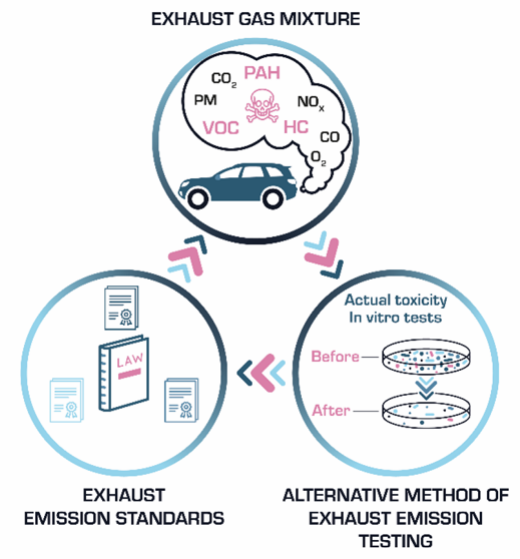

Most of the current studies on vehicle engine exhaust emissions are focused on qualitative and quantitative measurements. Approval tests for admitting vehicles to traffic and tests performed at vehicle inspection stations are limited to measuring the concentrations of individual compounds or selected groups of compounds. For vehicles with compression-ignition engines, the annual emission control comprises only an exhaust gas opacity test, performed with an opacimeter. This approach does not consider very harmful groups of compounds that determine the toxicity of exhaust gases but are not directly covered by the emission standards, such as polycyclic aromatic hydrocarbons and volatile organic compounds. Also, it does not provide a clear answer to the question of the actual toxicity of exhaust gases, understood as the harmful effect that a given substance causes on living organisms or biological processes. Studies on the actual toxicity of engine exhaust gases present a new area of interest, increasingly more discussed but still not approached in a comprehensive way. The studies include experiments using *in vitro* biological methods and chemical analyses of gas mixtures. In this Review, I present an overview of current research and a critical comparison of commonly used methods of testing engine exhaust emissions and methods that might supplement them in a significant manner. The development of *in vitro* biological methods, including methods of microscopic analysis of cells in the assessment of exhaust gas toxicity, provides an innovative approach to the problem of air pollution. This type of research presents the opportunity to indisputably answer the question of the actual toxicity of a given gas mixture and to make a new contribution to science in the field of molecular biology. Current data show that the survival of cells exposed to engine exhaust emissions from older generation vehicles is higher compared to that of newer generation vehicles.

## 1 Introduction

Based on the principle of sustainable development, promoted in Europe since the beginning of the 19th century, economic development should lead to improvement in quality of the natural environment, for example, by limiting the harmful impact production and consumption pose on the environment and by protecting the natural resources. According to the World Health Organization, in 2019, 99% of the world population had been living in places where the level of air pollution was higher than is considered acceptable.^1^ Air pollution poses a problem, especially in cities with increasing population density. One of the main sources of air pollutant emissions, threatening the natural environment, human health, and lifespan, is road transport. In central districts of large cities, especially ones with a centralized heating system, the share of road transport in the total emission of pollutants reaches 90%.^2^ Despite the fact that the air quality has been slowly improving over the years, air pollution remains one of the most serious environmental threats to human health, reducing the quality of life through disease and causing an estimated half million premature deaths among Europeans each year.^3^

It is important for modern engineering to take urgent action both to effectively reduce the current pollutant emission levels and to sensibly transform at least the production technologies responsible for the most burdensome and large-scale pollution. Almost immediately after the introduction of vehicles with internal combustion engines, the pursuit to minimize the harmful impact of exhaust gases on the environment became a trend in the global automotive industry. In Europe, this resulted in the adoption of directives, which introduced increasingly stringent European emission standards for commercial vehicles. Over the last 30 years, the standards that define permissible levels of harmful combustion products have undergone many changes. Every year, vehicle manufacturers face more and more construction and technological challenges, aimed at meeting the requirements and allowing vehicles to be placed on the market. Therefore, it should be constantly considered whether the adopted standardization is accurate, properly targeted, and effective and if it proves successful in improving our health and the nature that surrounds us.

**Figure 1:**
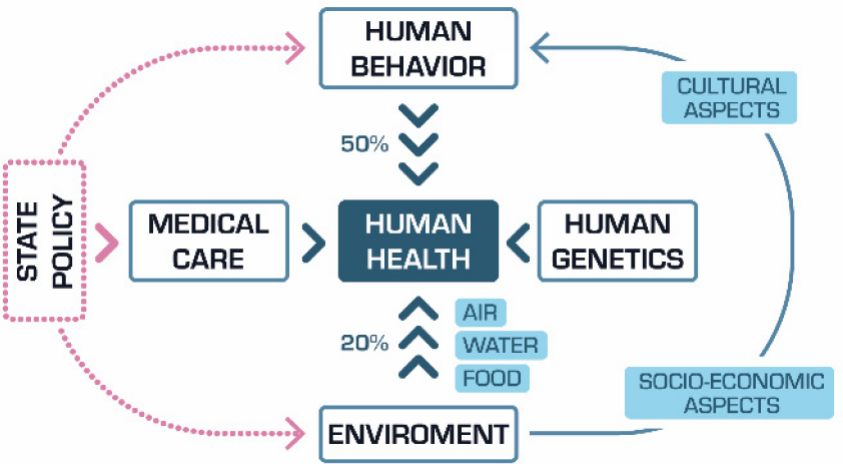
Factors affecting human health.

The policy of a given country has a significant impact on the health of its inhabitants. The level of healthcare, as well as the condition of the environment and human behavior, are largely determined by the policy applied in a country (Figure 1).

Among all factors affecting human health, human behavior (for example: addictions, bad eating habits) has the largest (50%) impact. Another important group includes biological, chemical, and physical factors (for example: diseases, radiation, noise, vibrations) that affect human health through the quality of air, water, and food. Their impact is estimated at 20%. The impact of other factors, for example human genetics and medical care, is estimated at around 30%.^4^

Public health should not be the price to pay for industrialization without sufficient environmental protection measures.

Despite the dynamic development of electromobility, internal combustion engines as a source of energy will remain an important issue for many years, especially in the context of the analysis of the emitted gases and their impact on living organisms. The current decline in economies of many countries is causing a significant decrease in sales of modern, expensive vehicles, including vehicles with electric drive. According to automotive industry analysts, the situation in the coming years will promote the rental of young used vehicles and increase the demand for repair and maintenance services.^5^ The interest in biofuels, which have the potential to significantly reduce levels of carcinogenic pollutants,^6,7^ is also expected to increase. Therefore, studies on the emission of harmful compounds from internal combustion engines and their impact on human health remain important topics that require further development.

A very important aspect in the development of internal combustion engines is the reduction of pollutant emissions. The toxicity of exhaust gases is determined mainly by the polycyclic aromatic hydrocarbons (PAHs) contained in them, which belong to unregulated emissions.^8^ The current regulations of the European Union regarding exhaust gas emission testing regulate only the permissible level of concentration of the entire group of hydrocarbons. Therefore, methods of testing the emissions of individual compounds from the group of hydrocarbons are not used in common practice. Therefore, the actual toxicity of engine exhaust fumes is unknown.

In the current literature, there is insufficient information about the existing methods for assessing unregulated emissions and proposals for their further development. Below, I present an overview and comparison of commonly used methods of measuring engine exhaust emissions based on qualitative and quantitative tests of harmful compounds. In the later part of this review, I present the methods of testing the actual toxicity of engine exhaust gases that are not commonly used in practice. The aim of the analysis is to indicate the advantages and disadvantages of legally regulated engine exhaust emission measurement methods. In addition, the analysis points out the directions for the development of methods, which could improve reliability of the quality assessment of exhaust gases and thus clearly determine the toxic effect of exhaust gases on a living organism.

## 2 Regulated and Unregulated Emission

The popularity of qualitative and quantitative tests of harmful compounds emitted from internal combustion engines comes from the need to comply with statutory emission standards, which contain permissible concentration values of individual compounds or groups of compounds. In Euro standards, in force in the European Union countries (Table 1), the limits of permissible concentrations are set collectively for the entire groups of hydrocarbons (HC) and nitrogen oxides (NO_*x*_) as well as for carbon monoxide (CO) and particulate matter (PM). In addition, the standards limit the amount of emitted particulate matter (PN). Today, almost 70% of light and heavy vehicles sold worldwide meet the current Euro 6 or the former Euro 5 standard.^9^

**Table 1:**
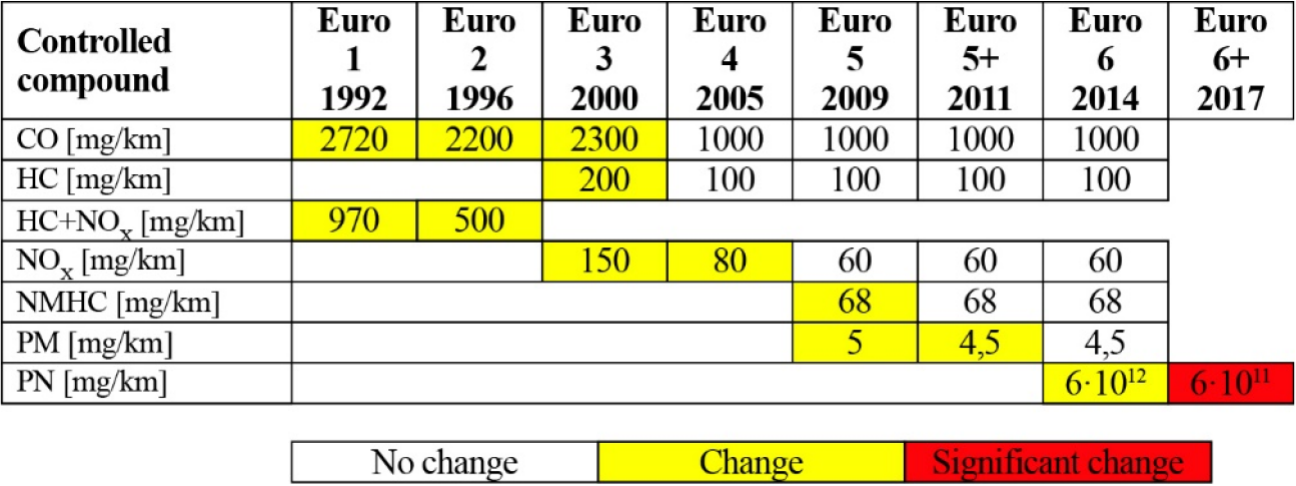
Emission Limits for New Passenger Vehicles Equipped with Gasoline Engine^10^

**Figure 2:**
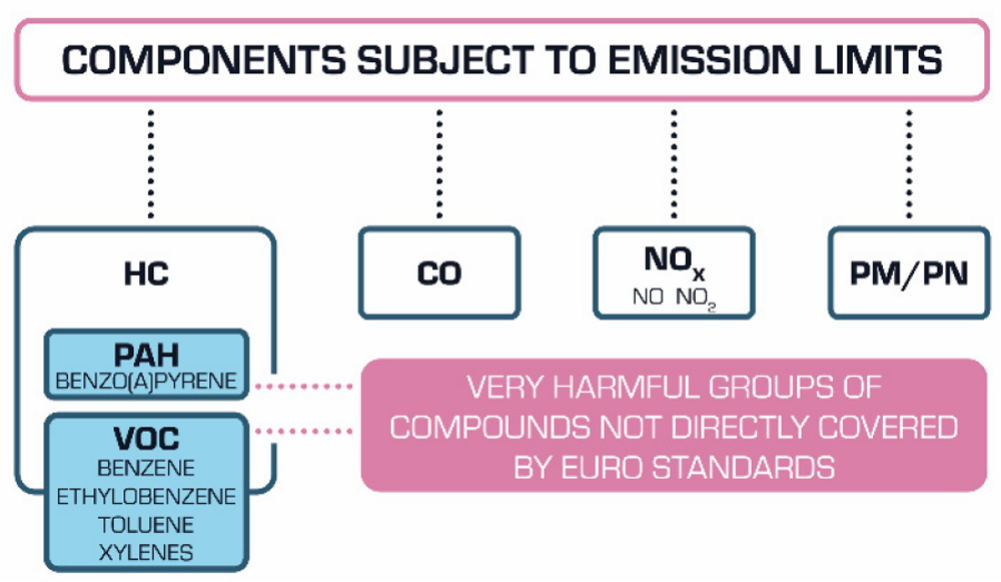
Most important toxic components of exhaust gases emitted from vehicles.

However, it has been scientifically proven^11^ that the actual toxicity of engine exhaust gases is determined by the hydrocarbons. Among hydrocarbons, we can distinguish from several dozen to several hundred different compounds which, when present in relatively small amounts, often cause carcinogenic, mutagenic, or genotoxic reactions^12^ and their toxicity may be additionally intensified by the phenomenon of additive synergism. When there are several substances in the atmosphere, their combined effects can be equal to the sum of their individual effects but can also be much stronger. The combined toxicity of compounds in a group of hydrocarbons can increase up to a thousand times compared to the sum of their individual toxicities, which is indicated by the values of the maximum permissible concentrations (TWA)^13^ and toxicity factors.^14^ Therefore, reducing the concentration of hydrocarbons in exhaust gases does not affect their actual toxicity. The most harmful groups of compounds among hydrocarbons include volatile organic compounds (VOCs) and polycyclic aromatic hydrocarbons,^15^ which are responsible for increasing incidences of asthma and irritation of mucous membranes and causing allergies and cancer (Figure 2).^16^ VOCs also contribute to ground-level ozone concentrations, particularly in megacity regions.^17^ Attention should be paid to the particularly toxic narcotic gases: among the VOCs the aromatic hydrocarbons, such as benzene, toluene, ethylbenzene, and xylenes (BTEX group) and among the PAHs the highly carcinogenic benzo(*a*)pyrene.^18^ Considering the current state of knowledge, it might be worth proposing changes in legal regulations to reduce the emission of additional compounds, extremely important from a toxicological point of view. Studies have proven that various exhaust gases, despite meeting the limits of the current emission standards, present significant,^19^ differentiated^20^ toxic effects.

Unlike the group of hydrocarbons, nitrogen oxides, including nitric oxide (NO) and nitrogen dioxide (NO_2_) as acid gases, and chemically asphyxiating carbon monoxide, are directly covered by emission limits and well recognized in terms of toxicology. However, current regulations on the emission limits of toxic compounds define the measurement of nitrogen oxide emissions as the volumetric concentration of NO_*x*_, and the mass emission is calculated by multiplying the given value by the NO density, despite the low content of this compound in NO_*x*_. In places of traffic congestion, an increase in the NO_2_ concentration in the air is recorded, despite the decrease in the NO concentration. This presents a serious problem, since NO_2_ is significantly more toxic than NO.^21^ It also raises questions about the correctness of the current methodology for controlling engine exhaust emissions. The distinction between NO and NO_2_ is not relevant from a vehicle-type-approval point of view, but it is important from an air pollution perspective.^22^

## 3 Exhaust Emission Test Methods

In recent years, the main purpose of qualitative–quantitative measurements in research on exhaust gas emissions conducted around the world is the testing of driving cycles under real traffic conditions.^23,24^ In addition, the studies are focused on testing new fuel mixtures^25,26^ and systems of exhaust gas treatment.^27,28^ Many publications also describe tests aiming to confirm compliance with emission standards.^29^ The researchers less frequently focus on secondary emissions of toxic compounds.^30^ The aforementioned research directions are based on various methods of measuring exhaust gas emissions, including exhaust gas analyzer-based methods, calculation methods, and analytical methods. These methods are suitable for testing exhaust emissions under stationary conditions on a chassis dynamometer or under traffic conditions.

The most common method used for measurements under real vehicle traffic conditions is based on the use of the PEMS (portable emissions measurement system) mobile research equipment, installed in the vehicle.^31−33^ It is used during the tests carried out before the vehicles are admitted to the market. The concentration of individual pollutants, the traffic energy, and the mass flow rate of the drive unit exhaust gases are measured at the same time. The apparatus can be used to test vehicles of various types and homologation categories. The values of the measured parameters are obtained immediately, which allows us to quickly draw conclusions from the conducted research. Unfortunately, preparing the PEMS apparatus for testing, keeping it ready, and carrying out the measurements requires a lot of effort from the user. First, the installation of the apparatus requires adjusting its elements to the exhaust system of each tested vehicle. It is especially challenging in vehicles not equipped with a towing hook. The system operates under strictly defined conditions, so the equipment needs to be conditioned before the measurements can be started. Communication between components is easily disturbed, and the process of recording and processing data is complicated even for an experienced user. In addition, the equipment requires frequent calibration with expensive technical gases and frequent filter changes. The users complain about frequent failures, high service costs, and long repair times. It is also important to remember that the exhaust gas analyzers included in the apparatus are subject to mandatory cyclical metrological inspections. Due to many inconveniences when using the PEMS apparatus, it might be worth considering supplementary or alternative solutions.

A common way of measuring pollutant emissions under stationary conditions on a chassis dynamometer is provided by simple exhaust gas analyzers,^34^ which use infrared radiation to measure the concentration of compounds, flame ionization analyzers, which measure the levels of hydrocarbons and methane, and chemiluminescence analyzers, which measure the concentration of nitrogen oxides. They are used primarily at vehicle inspection stations to carry out annual inspections of the quality of the emitted exhaust gases in vehicles equipped with spark-ignition (SI) engines. These analyzers are designed to measure the levels of the exhaust gas components, such as carbon monoxide, carbon dioxide, nitrogen oxides, hydrocarbons, and oxygen. Methods based on exhaust gas analyzers (including PEMS) are used to test the concentrations of individual compounds or the total concentrations of groups of compounds, covered by the emission standards. In Poland, exhaust gas analyzers are subject to legal regulations, which impose certain obligations related to their trade and use. In addition to the mandatory initial verification and reverification every 6 months, calibration of indications and periodic replacement of the oxygen sensor are required. The equipment of the vehicle inspection station also includes opacimeters, which are used to measure the opacity of exhaust gases for vehicles equipped with compression-ignition (CI) engines. Opacimeters are not subject to reverification and require only periodic mechanical calibration. The annual emission control is therefore clearly limited for CI vehicles, which still account for a significant 37% share among all vehicles registered in Poland.^35^ The exhaust gases emitted from CI engines are considered to be more harmful to human health than exhaust gases emitted from SI engines. In 2022, a decade has passed since the World Health Organization officially recognized the exhaust gases emitted from CI vehicles as carcinogenic. It is also important to note that commercial NO_*x*_ analyzers do not measure the actual concentration of NO_*x*_, as the measurements are disturbed by other reactive forms of nitrogen (NO_*y*_), mainly nitrous acid, nitric acid, nitric acid anhydride, and other relevant air pollutants. As a result, the values of the NO_*x*_ concentrations are measured with a large and often fluctuating deviation. Test results indicate that the NO_*x*_ concentration value constitutes on average less than half of the total NO_*y*_^36^ concentration.

When methods of testing exhaust gas emissions are described, it is necessary to mention the calculation methods. Those methods are used to compare the toxicity of gas mixtures with known qualitative and quantitative compositions, where all components are standardized compounds. Although the computational indicators have been used by researchers for years, for example to assess pollutant emissions in landfills^14^ or to assess engine performance and exhaust emission parameters in CI engines,^37^ they have not been put to commercial use. Toxicometer measurements are an adequate indicator of the toxicity of a gas mixture if it is possible to find a reference compound that can represent a given gas sample and reliably reflect the degree of toxicity of the tested mixture. Therefore, the choice of the reference compound should be strictly dependent on the qualitative and quantitative composition of the tested mixture and should consider the properties of its individual components.^10^

Analytical methods, including chromatographic and spectroscopic methods, are often used to measure exhaust emissions in scientific research. Those methods include the popular gas chromatography coupled with mass spectrometry (GC-MS),^38−40^ UV–vis absorption spectroscopy,^41^ and spectroscopy combined with remote sensing technology.^22^ They are used not only for identification of standardized compounds, including particulate matter,^42^ but also for the identification of compounds from the VOC^43^ and PAH^44^ groups.

Recent studies show that we have yet to develop a comprehensive characterization of PAH emissions from vehicles due to the current limitations of analytical methods. Therefore, the known PAH emission levels may be largely underestimated.^45^ Also, the comprehensive characterization of VOCs has been an analytical challenge^46^ for a long time. Currently, researchers are working on modifications of conventional analytical methods, including gas chromatography. Introducing new hardware configurations, based on the use of two cooperating chromatographic systems, guarantees the extension of compound detection and quantification limits.^47^ The use of analytical techniques in exhaust gas emission tests is an effective tool for the assessment of emitted organic compounds. However, these methods are subject to significant error. The average error of VOC or PAH quantification methods oscillates in the range of 25–30%.^42^ While the operation of modern chromatographs is considered highly reliable, the method requires a difficult and tedious preparation of samples for analysis. The sample preparation is the most important step of the test process, due to its largest contribution to the overall error of the analysis.^48^ Nevertheless, gas chromatography remains the only existing method for the qualitative and quantitative analysis of hydrocarbon compounds.

The emission limits defined by the current Euro standards are more than 1 order of magnitude lower than the emission limits specified in the original regulations. At the same time, they cover a wider set of pollutants and testing conditions. Therefore, it is important that the results of engine exhaust emission tests obtained using different methods are similar, as this proves the effectiveness and practicality of the applied methods. An exemplary analysis is the comparison of the results of gaseous emission tests from engines of CI vehicles, obtained using a PEMS apparatus and using remote sensing devices.^49^ By verifying a given method with an alternative method, it is possible to confirm the credibility of existing discrepancies between the actual emissions from vehicles under road conditions and during the type-approval laboratory test procedure.^9^

The current Euro 6 emission standards for light and heavy vehicles were first introduced almost a decade ago. Although the testing mechanisms were updated in the years following the introduction of the standard, the limits remained unchanged. It is worth to mention that the quality of emissions from combustion vehicles improved only in 2017, after the introduction of regulations on the RDE (real driving emissions) tests.^50−52^ In 2019, the European Commission initiated a regulatory process to increase the stringency of emission standards and introduced Euro 7, which meets the requirements of the European Green Deal.^53^ The prepared standard introduces no changes to the testing methodology but further tightens the concentration limits for harmful compounds. It is a known fact that the current tests do not cover the full range of frequent driving conditions that can result in increased emissions, such as short urban distances of less than 16 km, ambient temperatures below −7 °C or above 35 °C, or altitudes above 1300 m. Difficulties in emission control also result from the regulation loopholes, which create opportunities to optimize emission control systems in vehicles in order to improve the type-approval test results.^54^ Another problem is the fact that the standards do not limit the emissions of some of the very harmful groups of compounds that determine the toxicity of engine exhaust gases.^55^

**Figure 3:**
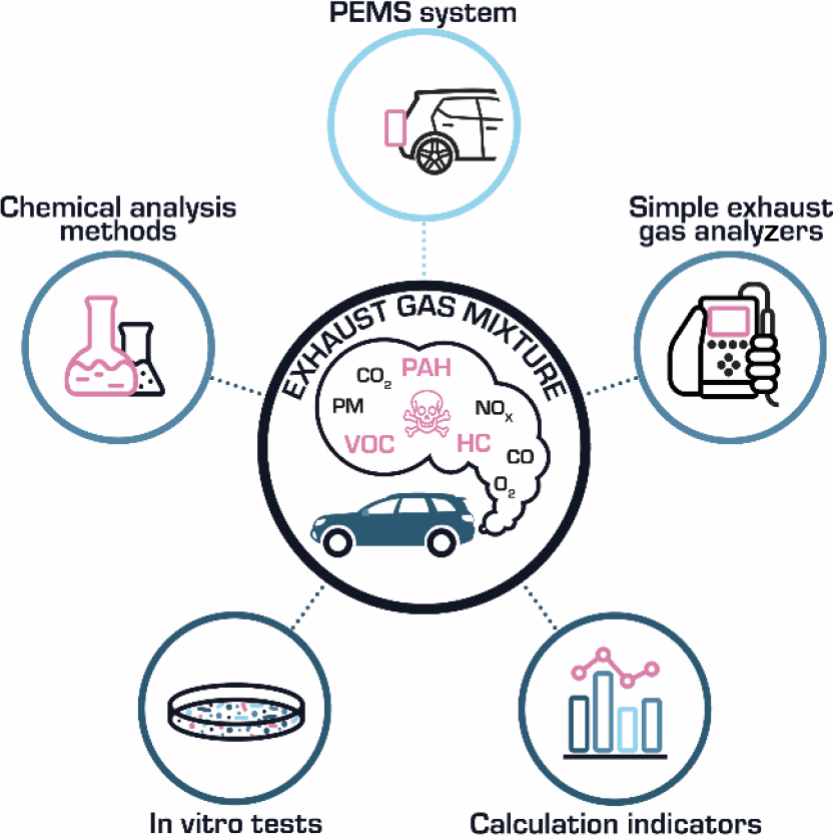
Methods of measuring exhaust gas emissions from vehicles.

The emission standards in their present form certainly raise some questions. At the same time, there are alternative solutions that can be used to determine the actual toxicity of engine exhaust gases in a simple and undisputed way, reflecting the actual impact of exhaust gases on living organisms (Figure 3). For at least several years, biological methods that use *in vitro* tests have been developed to assess the cytotoxicity and morphology of cells exposed to engine exhaust gases. Cell lines of the lungs, bronchi, or epidermis are usually selected for testing to imitate the ways pollutants are absorbed into the body. Studies on living cells are based on several methods that are commonly used to assess cellular cytotoxicity through observation of changes in the integrity of the cell membrane, changes in the activity of lysosomes, activity of enzymes related to cell metabolism, the ability of the cell to divide (proliferation), and activation of programmed cell death pathways. In addition, thanks to microscopic methods, with properly fixed samples, it is possible to observe morphological changes occurring in cells after exposure to toxins.

The precursor of research in which living cells are exposed to pollutants inhaled by humans is the German company Vitrocell,^56^ which has been focusing on the development and production of *in vitro* systems for over 20 years. Their research is currently focused on evaluating the cytotoxicity of combustible tobacco products.^57^ The company is also experienced in the assessment of toxicity of gases emitted from CI engines.^56^ Numerous research on the actual toxicity of engine exhaust gases have been performed for the air immissions in urban agglomerations^58^ and the interior of vehicle cabins.^59^ Studies of engine exhaust emissions toward the actual toxicity have been more widely undertaken by Swiss scientists.^60^*In vitro* research on engine exhaust emissions is also conducted in Italy,^61^ Poland,^55^ USA,^62^ Columbia,^63^ and China.^64^ It is worth taking a closer look at the methodology of research conducted using living cells to assess the progress of the work and identify research gaps that may contribute to the development of alternative methods for unambiguously determining the actual impact of exhaust gases emitted from vehicles on living organisms. The key issue in the study of cell cytotoxicity is not the selection of the appropriate test. It is the selection of appropriate parameters of cell exposure to harmful substances and learning the dose of the tested gases that causes the toxic effect.

**Figure 4:**
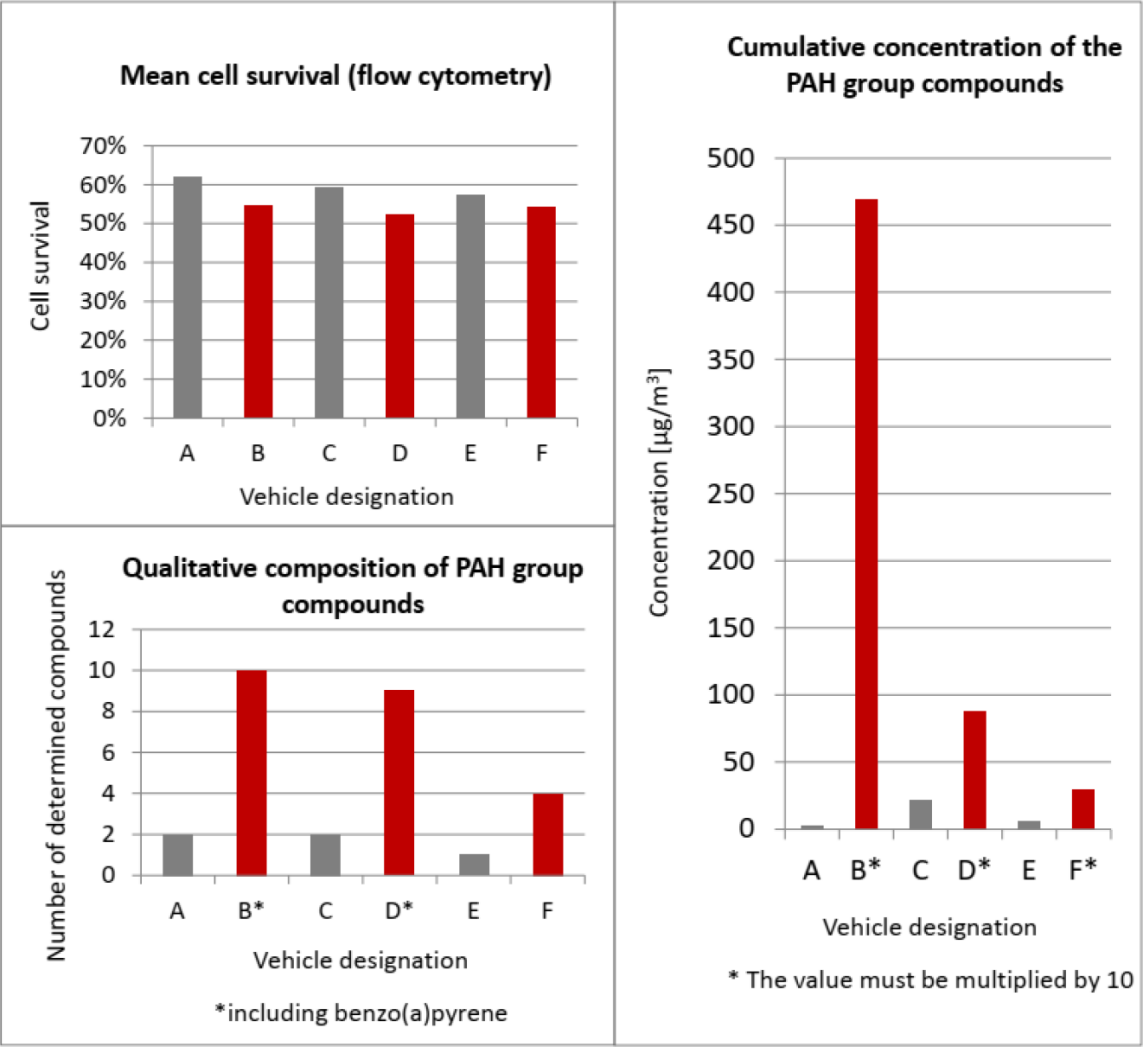
Correlations between the values of percentage cell survival and the number and cumulative concentration of compounds determined in the PAH group.^55^

In the studies performed by the Swiss team, morphological changes and cytotoxicity were observed in the cells of the 16HBE14o line after 6 h and 3 × 6 h exposure to 10-fold-diluted exhaust gases emitted from a CI vehicle. It should be noted that during the test, the cells were immersed in culture fluid, providing a barrier from the direct impact of the tested gases. The results have shown that only long exposure to exhaust gases causes an increase in the expression of proinflammatory genes.^60^ In turn, researchers in Poland performed cytotoxicity assessment of the L929 cell line, which was subjected to a short 7.5 min exposure to exhaust gases from SI vehicles without the use of culture fluid in a BAT-CELL device. It is currently an original solution for the methodology of this type of research. Studies have shown that Euro 6 vehicles have 4% higher cell survival rate than Euro 3 vehicles^55^ (Figure 4). Therefore, selecting the appropriate time of cell exposure to toxins is very important in the measurement procedure and should be adjusted depending on the use of physicochemical barriers that hinder direct contact of cells with the toxic gas mixture.

The studies of the remaining groups were based only on the characteristics of particulate matter emitted in the exhaust gases of SI or CI vehicles. The test cell lines were placed in a suspension of solid particles for 24 h. The Italian study revealed a slight decrease in cell viability after exposure to particulate matter from a Euro 3 vehicle compared to a Euro 6 vehicle.^61^ All research groups have confirmed that particulate matter activates proinflammatory and carcinogenic pathways in the body.^61−64^ In addition, studies in China have shown that particulate matter damages the cell membrane.^64^

Cell cytotoxicity studies have usually shown a correlation with the presence of PAHs in engine exhaust gases.^55,61,63^ It has been shown that the composition of hydrocarbons is related to changes in the number of cells after exposure to a mixture of exhaust gases emitted from tested passenger cars. The more diverse the qualitative and quantitative composition of PAHs and VOCs, the more cells degenerated.^55^

It is worth mentioning that microscopic methods are an important tool in the biological evaluation of cells. Confocal microscopy is used to assess cell morphology after exposure to gas mixtures,^60^ and transmission electron microscopy is used to assess the morphology and size of solid particles.^61^ It is possible to observe fluorescently stained cells, cells in culture fluid, frozen cells, and classically fixed cells. Thanks to microscopy, it is possible to follow the changes in the composition of the cell ultrastructure, the number of cells, and the dynamics of their movement. Such studies on cells exposed to exhaust gases have not yet been undertaken, as they require basic research in the preparation of microscopic slides appropriately for each cell line tested.

## 4 Development Prospects for Nonstandard Methods

*In vitro* methods, unlike the other common exhaust gas analyzer-based and analytical methods, allow observing the effect of interaction between compounds. For example, PAHs reacting with NO_*x*_ form nitro-PAHs,^65^ which would not be included in the research conducted with other methods. In addition, *in vitro* methods are not sensitive to calculation factors, dependent on changing legal regulations. The assessment of the exhaust gas mixture is carried out in a holistic manner, without the need for knowledge about the qualitative and quantitative composition of its individual compounds. The experiment is relatively easy to perform, and the sampling itself is noninvasive, unlike, for example, emission measurements performed using the PEMS system. The cell lines used in the research are commercially available. Therefore, there is no need to obtain the approval of the Bioethics Committee or the Local Ethical Committee for Animal Experiments. The time of cell exposure to the toxic mixture of exhaust gases can be reduced to minutes. Simple cytotoxicity evaluation, including determination of cell viability, requires only placing the cell solution in an automatic counter. In addition to the low cost of experiments and short testing time, the undoubted advantage of *in vitro* methods is the unambiguity and indisputability of the results. The percentage of cells that have degenerated after exposure to a given mixture of exhaust gases allows for a quick assessment of their toxicity.

Exhaust emission measurement methods based on the use of analyzers do not provide sufficient control of the emission of toxic compounds. Their common use is a result of specific legal regulations and standardization of selected compounds in the exhaust gas mixture. The use of analytical methods in the assessment of emission is substantial, as they provide a way to control almost all substances, including hydrocarbons, which are a very numerous and diverse mixture of harmful and toxic compounds that determine the toxicity of exhaust gases. Quantifying more compounds during vehicle approval would require additional chemical analyses and the definition of concentration limits for further compounds. However, such supplementation would be highly recommended to improve control of human exposure to toxins.

In the near future, *in vitro* biological methods seem to provide an effective and reliable solution to the current problems and controversies related to the present methods and legal regulations for testing the actual toxicity of vehicle exhaust gases. To introduce the method to general use, existing research procedures should be standardized, including methods of noninvasive and automatic gas sampling under real traffic conditions,^63^ conditions for exposing cells to toxic gases, and selecting an appropriate cytotoxicity assessment test. In addition to improving the methodology of testing the actual toxicity of engine exhaust gases, by popularizing the *in vitro* methods, it is possible to gain new knowledge about cellular mechanisms. This includes an integral analysis^66^ of the course of the cell cycle and apoptosis in cells, which occurs during the cell exposure to various types of air pollutants. The knowledge in this area is still insufficient.^67^ While knowledge about the chemical and mutagenic properties of pollutant components such as PAHs is currently quite extensive,^68−70^ little is known on the cellular factors involved in cellular responses to pollutants. At the same time, cellular and molecular biology events occurring during toxin poisoning provide a medical basis in favor of developing better legal solutions leading to the reduction in the actual emission of toxic compounds. The development of new test methods should therefore be based on the knowledge about the molecular biology underlying emission-induced pathology and be used by health legislators to prevent introduction of legislations that do not directly affect human health.^67^

## 5 Conclusions

Current data show that the introduction of increasingly stringent legal regulations does not go hand in hand with improving the quality of atmospheric air. New vehicles meet the set emission limits, but tests of the actual toxicity of exhaust gases indicate only statistically insignificant differences in the quality of emitted exhaust gases for newer and older generation vehicles. This is mainly due to PAH compounds, which are not subject to a standard assessment. Therefore, the introduction of increasingly more stringent emission standards for commercial vehicles in its current form requires careful analysis on the legislators’ side. Extending the number of limited compounds (research using gas chromatography) and introducing *in vitro* tests as a supplementary method of exhaust gas mixture analysis that reflects the synergistic effects of compounds might prove to be a credible solution to make a real impact on the condition of the environment. The development of *in vitro* biological methods, and with them the methods of microscopic analysis of cells in the assessment of exhaust gas toxicity, means undertaking work on the application of existing measurement methods for testing the quality of exhaust gases emitted from engines. This is an innovative approach to the problem of air pollution that indisputably answers the question of the actual toxicity of a given gas mixture. Moreover, this work will constitute a new contribution to science in the field of molecular biology.
